# Scaling Adolescent Immunity: Multi-Age Cohort Human Papillomavirus (HPV) Vaccination Campaigns in West Africa—Evidence from Côte d’Ivoire, Ghana, Liberia, and Sierra Leone

**DOI:** 10.3390/vaccines14090728

**Published:** 2026-08-24

**Authors:** Ado Mpia Bwaka, Sambo Guemgo, Marcellin Mengouo Nimpa, Pamela Mitula, Hermann Didi Ngossaki, Sylvain Honore Woromogo, Hadiatou Diallo, Milse William Nzingou Mouhembe, Crépin Hilaire Dadjo, Annick R. Ayele Dosseh, Edinam Agbenu, Lynda Rey, Akpaka A. Kalu, Benido Impouma

**Affiliations:** 1 World Health Organization, Regional Office for Africa, Inter-Country Support Team for West Africa, Ouagadougou 01, Burkina Faso; sambo.guemgo@alumni.emory.edu (S.G.); nimpamengouom@who.int (M.M.N.); mitulap@who.int (P.M.); sworomogo@who.int (S.H.W.); diallohad@who.int (H.D.); milse.nzingoumou@who.int (M.W.N.M.); dadjoh@who.int (C.H.D.); dosseha@who.int (A.R.A.D.); agbenue@who.int (E.A.); 2 World Health Organization, Regional Office for Africa, Brazzaville P.O. Box 06, Congo; lynda.rey@who.int (L.R.); kalua@who.int (A.A.K.); impoumab@who.int (B.I.)

**Keywords:** HPV vaccine, multi-age cohort (MAC), campaign, coverage, assessment

## Abstract

**Background**: Cervical cancer remains a major public health problem, causing approximately 660,000 new cases and 348,000 deaths annually, with more than 90% occurring in low- and middle-income countries. The WHO African Region accounts for approximately 20% of global cases and 30% of deaths. In November 2020, the World Health Assembly adopted the Global Strategy for Cervical Cancer Elimination with 90–70–90 targets for 2030. Multi-age cohort (MAC) campaigns have emerged as a high-impact strategy for achieving population-level HPV vaccination coverage. This study documents the implementation and outcomes of HPV MAC campaigns across Côte d’Ivoire, Ghana, Liberia, and Sierra Leone in 2025. **Methods**: This multi-country mixed-methods analysis integrated quantitative administrative campaign data with qualitative programmatic assessments across eight operational readiness domains from the WHO Immunization Readiness Assessment Tool. Data were collected between January and December 2025 from national reports, WHO supervision missions, and partner reports. Coverage analysis used administrative data triangulated with independent monitoring. Equity analysis used school enrolment data and geographic accessibility indices. **Results**: The campaigns targeted 7,376,096 girls aged 9–18 years and reached approximately 6,306,294, achieving 85.5% overall coverage. Côte d’Ivoire, Ghana, Liberia and Sierra Leone reported administrative coverage of 88.0%, 84.5%, 60.0% and 100%, respectively. All four countries achieved the minimum operational readiness threshold of 80% across all assessed domains. A total of 35,860 health workers were trained, with post-training competency scores increasing from 58.8% to 89.5%. Cold chain functionality exceeded 98% across all campaigns. A total of 314 cases of Adverse Events Following Immunization (AEFIs) were reported, all classified as mild, giving an approximate rate of 5.0 per 100,000 doses. Coverage gaps between in-school and out-of-school girls ranged from 18 to 35 percentage points. **Conclusions**: The findings suggest that large-scale adolescent HPV immunization can be successfully implemented in resource-limited settings when supported by political commitment, multisectoral coordination and adequate operational resources. The single-dose schedule transition under Gavi 6.0 offers opportunities to simplify delivery. Priority actions include integrating HPV into routine immunization, scaling equity strategies for out-of-school girls and ensuring sustainable financing.

## 1. Introduction

Cervical cancer represents one of the leading causes of preventable mortality among women worldwide. It is estimated to have been responsible for 660,000 new cases and 348,000 deaths in 2022, of which over 90% occurred in low- and middle-income countries [1]. The WHO African Region bears a disproportionate burden, accounting for approximately 20% of global cervical cancer cases and 30% of related deaths [2]. West Africa specifically records among the highest age-standardized incidence rates globally, with Guinea (50.1 per 100,000 women), Liberia (40.8 per 100,000), and The Gambia (42.9 per 100,000) ranking among the most affected [1].

Persistent infection with high-risk human papillomavirus (HPV) types, particularly HPV-16 and HPV-18, is responsible for more than 95% of cervical cancer cases [3]. In November 2020, the World Health Assembly adopted the Global Strategy for Cervical Cancer Elimination, establishing the 90–70–90 targets to be achieved by 2030: 90% of girls fully vaccinated with HPV vaccine by age 15, 70% of women screened by age 35 and again by age 45, and 90% of women with identified disease receiving treatment [4]. Achievement of these targets will avert over 74 million new cervical cancer cases by 2120 [5].

In December 2022, the WHO’s Strategic Advisory Group of Experts on Immunization (SAGE) recommended a single-dose schedule for girls aged 9–14 years, subsequently adopted by Gavi, the Vaccine Alliance, under the Gavi 6.0 strategy mandating transition to a single dose by 2026 [6]. The adoption of a single-dose HPV vaccination schedule provides an opportunity for countries to expand target age groups while reducing operational complexity and delivery costs, thereby increasing the feasibility of large-scale MAC campaigns.

Gavi, the Vaccine Alliance, is a global public–private partnership established in 2000 to improve equitable access to vaccines in low and middle-income countries through vaccine financing, health system strengthening, and global vaccine market shaping to increase affordability and availability [7]. As part of its efforts to advance global immunization goals in IA2030, Gavi supports the introduction of new vaccines into national EPI programmes, including HPV vaccines. WHO encourages countries to use MAC campaigns to rapidly vaccinate target populations and older cohorts, as this accelerates achievement of the WHO targets of 90% HPV vaccination coverage [4,6,8].

Girls and adolescents living with HIV/AIDS require two to three doses as immunocompromised individuals may mount a suboptimal immune response to a single dose [6]. MAC campaigns have increasingly been adopted as a strategy to rapidly increase HPV vaccination coverage by reaching both routine target populations and older cohorts who may have missed vaccination opportunities [9].

Although several African countries have recently implemented MAC HPV vaccination campaigns, there is limited published evidence describing operational readiness, implementation strategies, coverage achievements, and lessons learned at scale across multiple countries in the WHO African Region. Most published studies focus on pilot introductions or single-country experiences, limiting opportunities for cross-country learning.

This article provides a comprehensive analysis of HPV MAC vaccination campaigns implemented across four West African countries during 2025. Côte d’Ivoire, Ghana, Liberia, and Sierra Leone represent diverse epidemiological, health system, and sociopolitical contexts, providing valuable comparative insights into campaign implementation at scale. The analysis aims to (i) document operational readiness and campaign planning processes; (ii) analyze implementation strategies, delivery modalities, and coverage outcomes; (iii) assess equity, safety, and logistical performance; (iv) identify best practices and innovations; and (v) propose strategic recommendations for sustaining and expanding HPV vaccination coverage across the region.

## 2. Materials and Methods

### 2.1. Study Design

This multi-country descriptive study assessed readiness, implementation, and early results of 2025 HPV MAC campaigns in Côte d’Ivoire, Ghana, Liberia, and Sierra Leone. All four countries used the WHO-recommended single-dose schedule.

The analysis combined a mixed-methods framework, integrating quantitative administrative data with qualitative programmatic assessments. Data were collected between January and December 2025.

All four countries implemented their HPV vaccination campaigns using the quadrivalent HPV vaccine Gardasil® (Merck Sharp & Dohme LLC, Whitehouse Station, NJ, USA), which targets HPV genotypes 6, 11, 16, and 18.

### 2.2. Conceptual Framework

The analytical framework was structured around eight operational readiness domains adapted from the WHO Immunization Readiness Assessment Tool: (1) strategic planning and microplanning; (2) cold chain and logistics; (3) vaccine management; (4) capacity building and training; (5) advocacy and communication; (6) supervision and monitoring; (7) AEFI surveillance; and (8) coordination and partner engagement. Each domain was assessed against standardized indicators with scoring thresholds (greater than or equal to 80% deemed campaign-ready) [10]. Table 1 summarizes the conceptual framework, key indicators and data sources.

### 2.3. Data Analysis and Quality Validation

Data were collected from national routine immunization databases, operational reports of MAC campaigns and WHO and Technical partners’ supervision mission reports, and post-introduction evaluation findings where available.

Coverage analysis used administrative data reported through national immunization information systems, triangulated where available, with independent monitoring reports. Equity analysis used proxy indicators, including school enrolment data, geographic accessibility indices, and urban–rural distribution of vaccination sites. AEFI data were collected through national pharmacovigilance systems using standardized case report forms. To enhance reliability, vaccination and operational data were systematically triangulated with other sources.

## 3. Results

### 3.1. Comparative Campaign Overview

The four HPV MAC campaigns implemented during 2025 shared common strategic frameworks while adapting to distinct country contexts, health system capacities, and target populations. Collectively, the campaigns targeted 7,376,096 girls aged 9–18 years and reached approximately 6,306,294 girls, representing an overall coverage achievement of 85.5% (Table 2). Côte d’Ivoire targeted the largest population (3,535,286 girls aged 9–18 years), reflecting the government’s commitment to maximizing coverage among all eligible cohorts. Ghana, Liberia, and Sierra Leone targeted girls aged 9–14 years in accordance with national EPI policies. All four countries used the WHO-recommended single-dose schedule.

### 3.2. Operational Readiness Assessment

Operational readiness was assessed using the WHO standardized readiness assessment tool covering eight domains. All four countries achieved the minimum 80% threshold across all domains. Aggregate readiness scores ranged from 88.9% (Ghana) to 100% (Côte d’Ivoire, Liberia, and Sierra Leone). The readiness assessment was conducted 4–6 weeks before the campaign launch, allowing adequate time for gap remediation (Table 3).

Ghana identified specific gaps in vaccine management (66.7%) and capacity building (86.0%) domains, requiring targeted remedial action including additional stock management training and refresher sessions for district supervisors. All other countries achieved greater than or equal to 92% across all individual domains. Côte d’Ivoire, Liberia, and Sierra Leone achieved perfect scores in seven of eight domains, reflecting intensive pre-campaign preparedness investments [11,12,13,14].

### 3.3. Country-Level Campaign Results

#### 3.3.1. Côte d’Ivoire

Côte d’Ivoire launched its nationwide HPV MAC campaign on 25 April 2025, targeting 3,535,286 girls aged 9–18 years across all 113 health districts. The expanded age range reflected the government’s commitment to reach all eligible cohorts. The campaign deployed 12,190 vaccinators across 17,448 vaccination sites, with 32% fixed centres, 47% schools, 10% outreach sites, and 11% community locations. By campaign conclusion, approximately 3,110,000 girls had been vaccinated, achieving 88.0% administrative coverage. Coverage exceeded 90% in 78 of 113 districts (69%), with the highest coverage in the Abidjan autonomous district (95%) and lowest in the northwestern border districts (72%).

The campaign operated on a national budget allocation of 2.1 billion FCFA and mobilized 22,399 personnel through 4040 vaccination teams. Côte d’Ivoire used a stratified mixed delivery approach with school-based sessions as the predominant mechanism, complemented by extensive outreach through mobile vaccination teams, temporary community posts, and door-to-door mobilization. Digital monitoring was enhanced through DHIS2 tracking, 4851 ODK-based supervisions, and Power BI analytics for real-time decision-making. A total of 155 mild AEFI cases were registered. Vaccine wastage was maintained at 3.2%, well within the acceptable threshold [11,12,13,14].

#### 3.3.2. Ghana

Ghana launched its nationwide campaign in October 2025, targeting 2,226,993 girls aged 9–14 years across all 261 districts. The campaign deployed 12,000 vaccinators across 14,000 sites, with delivery platforms comprising 20% fixed centres, 53% schools, 12% outreach sites, and 15% community locations. School-based delivery was prioritized given the high rate of girls’ enrolment in schools (approximately 85% net enrolment at primary level). Ghana vaccinated approximately 1,880,000 girls, achieving 84.5% administrative coverage. Coverage exceeded 90% in 186 of 261 districts (71%), with the highest coverage in the Greater Accra and Ashanti regions.

Ghana’s campaign featured sophisticated digital dashboard systems representing regional best practice, enabling real-time coverage visualization, automated data compilation, decision support analytics, and regional trend analysis across 16 regions. This digital infrastructure enabled identification of underperforming districts within 24 hours cycles. Urban challenges in Greater Accra included population mobility, school absenteeism, and information access gaps among transient populations. The campaign recorded 94 mild AEFI cases. Vaccine wastage was 4.1%. Ghana’s post-campaign coverage survey validated administrative data within 5 percentage points, indicating good data quality [12].

#### 3.3.3. Liberia

Liberia implemented its campaign in November–December 2025, targeting 746,523 girls aged 9–14 years across all 30 health districts. Liberia deployed 2500 vaccinators across 2334 vaccination sites, distributed equally across four platform types (33% each for fixed centres and schools, 17% each for outreach and community locations). The equal distribution reflected Liberia’s context of limited school infrastructure in rural areas. Liberia vaccinated approximately 448,000 girls, achieving 60.0% administrative coverage. While this fell below the 90% target, it represented an important accomplishment in a post-conflict, resource-constrained setting.

The campaign was characterized by high political visibility and intensive monitoring systems. A national public launch featuring high-level officials signalled political priority. Daily national review meetings analyzed coverage data and deployed immediate corrective actions. Early monitoring indicated rapid initial progress: by Day 2, 289,171 girls (39%) had been vaccinated; by Day 3, 448,353 girls (60%). The highest performing counties included Gbarpolu (51%), Lofa (45%), and Grand Gedeh (48%). Coverage varied considerably by county, ranging from 78% in Montserrado (the capital county) to 42% in remote southeastern counties. Vaccine wastage was 2.8%, the lowest among the four countries, reflecting meticulous stock management practices. Challenges included supply shortages, cold chain issues in remote areas, and heavy rainfall affecting team mobility [13].

#### 3.3.4. Sierra Leone

Sierra Leone implemented its campaign in November–December 2025, reporting an administrative coverage of 100%, the highest among the four countries. The campaign targeted 868,294 girls aged 9–14 years across all 16 districts. Sierra Leone deployed 3200 vaccinators across 2900 vaccination sites and implemented a two-round strategy, with Round 1 in November targeting 9–12 year olds and Round 2 in December targeting 13–14 year olds. The two-round approach allowed for intensive follow-up and defaulter tracing between rounds. All 16 districts achieved at least 95% coverage.

Approximately two-thirds of vaccinated girls were reached through school-based platforms, with one-third through community outreach. Sierra Leone deployed 10 medically equipped mobile clinic buses as a distinctive innovation providing enhanced safety preparedness, mobile service delivery to remote communities, and supervision platforms, particularly in the Eastern Province. Rigorous pre-implementation readiness assessment identified gaps in microplanning and communication materials; intensive technical support substantially improved readiness before launch. The campaign recorded 43 mild AEFI cases. Vaccine wastage was 3.5% [14].

### 3.4. Consolidated Campaign Results

Table 2 presents a consolidated summary of key performance indicators across all four campaigns. The combined reach of approximately 6.3 million girls represents one of the largest regional HPV vaccination efforts documented in sub-Saharan Africa to date. The proportion of districts achieving greater than or equal to 90% coverage varied from 27% in Liberia to 100% in Sierra Leone, reflecting the influence of health system capacity, target population size, and implementation strategy on outcomes.

### 3.5. Training and Capacity Building

Capacity building was recognized as a foundational element of campaign preparedness across all four countries. A total of 35,860 participants were trained through a standardized four-tier cascade model: 670 master trainers at the national level, 5300 district supervisors at the subnational level, and 29,890 frontline vaccinators at the implementation level (Table 4). Training modules covered HPV disease and vaccine introduction, single-dose schedule and age eligibility, vaccine administration and safety, cold chain management, data recording and reporting, AEFI recognition and reporting, interpersonal communication and management of vaccine hesitancy, and defaulter tracing and mop-up strategies [12,14].

Pre- and post-training assessments showed knowledge improvement, with average scores increasing from 58.8% to 89.5% across all four countries. Liberia employed the longest training duration (5 days), reflecting the need for additional competency reinforcement in a post-conflict context. Ghana’s lower pre-test scores (58%) could be due to the relatively limited HPV vaccination experience among health workers. Training completion rates were 100% across all countries [11,12,13,14].

### 3.6. Implementation Strategies and Delivery Modalities

All four countries employed mixed-delivery strategies combining school-based, fixed centres, outreach, and community-based platforms. School-based delivery served as the primary platform in Côte d’Ivoire (47% of sites) and Ghana (53%), reflecting relatively high school enrolment rates. In Liberia and Sierra Leone, delivery was more evenly distributed across platforms (33% fixed centres, 33% schools, 17% outreach, 17% community), reflecting the need to reach populations in settings with less developed school infrastructure [13,14].

Digital monitoring innovations enhanced adaptive management capacity. Ghana and Côte d’Ivoire utilized DHIS2 dashboards for real-time campaign monitoring, rapid identification of coverage gaps at district and subdistrict levels. Sierra Leone’s two-round strategy proved particularly effective, with Round 2 serving as a mop-up phase that captured girls missed during Round 1 and corrected data quality issues. Sierra Leone deployed 10 medically equipped mobile clinic buses to serve hard-to-reach communities, particularly in the Eastern Province [14].

### 3.7. Social and Behaviour Change Strategies

Social and behaviour change (SBC) strategies were central to campaign success, addressing vaccine hesitancy, generating demand, and ensuring community acceptance. All four countries implemented multi-channel SBC approaches combining mass media, community engagement, and digital platforms (Table 5). High-level political endorsement was secured in all countries through presidential launches and ministerial statements. Religious leader engagement was particularly important in Ghana, where the Christian Health Association and National Chief Imam’s office issued public endorsements [11,12,13,14].

Radio remained the dominant mass media channel, with campaign messages broadcast in 12–15 local languages per country. Côte d’Ivoire’s media campaign achieved an estimated reach of 8 million people. A total of 81,500 community mobilisers were engaged across the four countries. Social media campaigns reached an estimated 3.5 million parents and caregivers. SMS messaging campaigns were implemented in Ghana and Côte d’Ivoire. Proactive AEFI communication strategies were implemented in all countries, including pre-positioned media statements and rapid response protocols. No major misinformation incidents requiring crisis communication were reported [11,12,13,14].

### 3.8. Logistics and Vaccine Management

Cold chain and logistics management was a critical enabler of campaign success. HPV vaccine doses were procured through UNICEF Supply Division using Gavi co-financing arrangements. All countries received vaccine consignments at least four weeks prior to campaign launch. Cold chain functionality exceeded 98% across all campaigns, with Côte d’Ivoire, Liberia, and Sierra Leone reporting 100% equipment functionality. Vaccine wastage rates ranged from 2.8% (Liberia) to 4.1% (Ghana), all well within the WHO acceptable threshold of 10%. All four countries used auto-disable syringes and safety boxes for sharps’ disposal [11,12,13,14].

### 3.9. Monitoring and Supervision

Monitoring and supervision systems were established at all administrative levels. Daily reporting using standardized tally sheets with DHIS2 electronic platforms was implemented in Côte d’Ivoire and Ghana. Standardized supervision checklists covering vaccination quality, cold chain maintenance, data recording, and AEFI surveillance were deployed across all countries. Independent monitoring by WHO and partner staff provided objective assessment of campaign implementation process. Côte d’Ivoire conducted 4851 ODK-based supervisions with Power-BI analytics. Liberia’s daily national review meetings enabled rapid corrective action.

Data quality assessments conducted mid-campaign identified and corrected issues in real time [10].

### 3.10. Adverse Events Following Immunization

AEFIs were monitored through established national AEFI surveillance systems. Across all four campaigns, a total of 314 AEFI cases were reported from approximately 6.3 million doses administered, yielding an overall reporting rate of 5.0 per 100,000 doses. All reported AEFI cases were classified as mild and self-limiting, consisting primarily of injection site reactions (pain, redness, swelling) and systemic symptoms (fever, headache, fatigue). No serious adverse events or deaths were reported. The consistent AEFI reporting rate across countries (4.9–5.0 per 100,000) is consistent with the established safety profile of HPV vaccines and suggests functional AEFI surveillance systems [10,11,12,13,14].

### 3.11. Equity Analysis

Equity in HPV vaccination coverage was assessed as a critical indicator of campaign quality. All four countries demonstrated higher coverage among in-school girls compared to out-of-school girls. The difference ranged from 18 points in Sierra Leone (98% vs. 80%) to 35 percentage points in Liberia (85% vs. 50%). Sierra Leone achieved the narrowest difference. Coverage in hard-to-reach areas ranged from 55% (Liberia) to 78% (Sierra Leone).

Girls and adolescents living with HIV/AIDS require two to three doses of HPV vaccine as per WHO immunization guidelines for immunocompromised individuals. All four countries included provisions for identifying and appropriately vaccinating HIV-positive girls through linkages with antiretroviral therapy clinics. Data on coverage among HIV-positive populations were limited.

## 4. Discussion

### 4.1. Coverage Performance and Equity in Context

The combined MAC in four countries reached approximately 6.3 million girls, making it one of the largest regional HPV vaccination efforts documented in sub-Saharan Africa to date [11,12,13,14]. These results suggest that large-scale adolescent immunization is feasible in resource-limited settings when supported by strong political commitment, effective multisectoral coordination, and adequate technical and financial resources. This finding is consistent with other studies in low-income countries, including Nigeria and Rwanda [9,15,16].

The marked heterogeneity in campaign performance across the four countries provides important insights into the determinants of successful HPV vaccination scale-up. Sierra Leone’s exceptional coverage (100.1%) was achieved through its innovative two-round implementation strategy that enabled intensive mop-up and defaulter tracing between rounds, combined with strong community ownership fostered through 15,000 female mobilisers. Côte d’Ivoire (88.0%) and Ghana (84.5%) both approached the 90% WHO target despite their considerably larger scale, demonstrating that high coverage is achievable even with target populations exceeding 2 million girls when supported by a strong health systems and effective school-based delivery platforms [9,17]. Sierra Leone administrative coverage of 100.1% may be hiding inaccuracies in target population estimates, population movement, or vaccination of girls outside the originally projected target population.

Liberia’s 60.0% coverage, while below target, represents a commendable achievement in a post-conflict setting with human resource and infrastructure constraints. The 40.1 percentage-point gap between the highest (Sierra Leone) and lowest (Liberia) performers underscores the critical influence of contextual health system capacity, political commitment, and community trust-building on campaign outcomes. These findings align with broader evidence from sub-Saharan Africa indicating that fragile health systems, competing priorities, and sociocultural barriers impede sustained high coverage in resource-constrained settings [18,19,20].Equity analysis also revealed higher coverage among in-school girls compared to out-of-school girls, consistent with other studies [9,19], Low coverage in hard-to-reach areas ranged from 55% (Liberia) to 78% (Sierra Leone), reflecting geographic accessibility challenges, limited health infrastructure, and nomadic populations [10,20]. HPV coverage among HIV-positive populations who were eligible for a second dose of vaccination was limited. This could be explained by poor linkages and collaboration with antiretroviral therapy clinics, which need to be strengthened.

Despite the overall success of the campaigns, persistent inequities were evident. Across all countries, out-of-school girls were consistently less likely to be reached than girls attending school. This finding mirrors evidence from multiple African settings and highlights the limitations of predominantly school-based delivery models [9,15,16,19]. This also calls for integrating HPV vaccination within comprehensive cervical cancer prevention programmes and primary health care services [17,21]. Failure to address this population may compromise progress toward the WHO target of 90% HPV vaccination coverage.

### 4.2. Operational Readiness as a Determinant of Success

The observed alignment between operational readiness scores and campaign coverage outcomes suggest that the WHO standardized readiness assessment tool may be useful for timely identifying areas requiring corrective action before campaign implementation. All four countries that achieved greater than or equal to 88.9% aggregate readiness scores subsequently achieved greater than or equal to 60% coverage, with the three countries scoring above 99% all achieving greater than or equal to 88% coverage. Ghana’s lower readiness score (88.9%), driven primarily by gaps in vaccine management (66.7%) and capacity building (86.0%), was reflected in its comparatively lower coverage (84.5%) and the highest vaccine wastage rate (4.1%) among the four countries [10].

The timing of readiness assessments (4–6 weeks pre-campaign) proved adequate for gap remediation in all cases, suggesting that this interval should be considered a minimum standard for future MAC campaigns. The capacity building domain showed the greatest variability across countries, indicating that investment in training cascades should be prioritized early in the campaign planning process.

### 4.3. Best Practices and Innovations

Several best practices and implementation innovations emerged from this analysis and may provide useful lessons for future HPV vaccination in similar settings.

High-level political commitment, including presidential launches and ministerial endorsements across all four countries, may have contributed to enhanced public confidence and stakeholders’ engagement. Strong coordination between health and education sectors through formal memorandum of understanding and joint planning mechanisms facilitated school-based delivery and improved microplanning [10]. Similar successes have been reported in Rwanda, where strong political commitment and multicultural collaboration facilitated rapid HPV vaccine uptake [9,16].

Country-specific innovations also contributed to campaign performance. Liberia’s extended training duration (5 days) included systematic post-training assessment and daily coordination meetings. This strengthened accountability and required strong managerial support. Sierra Leone’s two-round strategy using medically equipped mobile clinic buses appeared effective in achieving near-universal coverage and reaching hard-to-reach communities. This may, however, increase operational costs. Ghana leveraged digital monitoring through DHIS2 dashboards, enabling real-time tracking of progress and identification of gaps. This also requires adequate internet and digital infrastructure at subnational levels. Côte d’Ivoire expanded mobile outreach activities to reach hard-to-reach and out-of-school girls, improving equity but requiring additional logistical and financial resources.

These findings collectively suggest that implementation strategies should be adapted to country context, health system capacity, and resource availability rather than applying a single approach across all settings. This is consistent with studies in WHO AFRO region [22,23].

### 4.4. Sustainability of HPV Programmes

Despite overall success, several persistent challenges were identified. The coverage gap between in-school and out-of-school girls (18–35 percentage points) suggests an equity challenge that school-based delivery models alone cannot address. This finding is consistent with global evidence demonstrating that HPV vaccination programmes disproportionately miss out-of-school and marginalized adolescent populations [9,15].

Sustaining these gains will require integration of HPV vaccination in routine immunization programmes, collaboration with school health programmes and maintaining outreach strategies for out-of-school girls. Côte d’Ivoire plans to maintain outreach strategies for out-of-school girls, while Ghana will leverage its digital monitoring systems and existing primary healthcare infrastructure. Liberia aims to strengthen community-based delivery and supportive supervision, while Sierra Leone will build on lessons learned from its two-round campaign model. Across all countries, continued political commitment, domestic financing, and partner support will be essential to sustain high coverage and advance cervical cancer elimination goals.

Vaccine hesitancy fueled by misinformation, particularly religious misconceptions linking HPV vaccination to infertility, was reported in all four countries. Similar trends were reported in Nigeria, Rwanda, and other settings [9,15,16]. Although the four countries currently prioritize young girls in line with WHO recommendations, countries could consider extending HPV Vaccination to boys in the future to reduce virus transmission and vaccine hesitancy as the programme matures and sustains high coverage among girls. Data quality and denominator issues affected performance tracking, with administrative coverage exceeding 100% in some districts indicating denominator inaccuracies. Human resource constraints, including high workforce turnover and competing routine immunization activities, created workload pressures.

Financial sustainability remains a concern, as all four countries remain eligible to Gavi financing for HPV vaccine procurement and campaign costs. The long-term sustainability of HPV vaccination programmes will increasingly depend on countries’ capacity to mobilize domestic resources as they approach different stages of Gavi transition [9,15,24].

Security-related access challenges in conflict-affected areas affected vaccination team deployment in border regions [10].

Table 6 summarizes the key implementation challenges, underlying causes, and mitigation strategies identified during the campaigns.

### 4.5. Limitations

This analysis has three main limitations. First, administrative coverage data may overestimate true coverage due to denominator uncertainties, particularly in low-income countries with incomplete civil registration systems and irregular national population censuses. Population-based coverage surveys or independent rapid monitoring and disaggregation of data coverage among people living with HIV could have provided more robust coverage estimates.

Secondly, data completeness varied across fragile and conflict-affected settings where programme monitoring systems are less developed. The analysis relied primarily on programmatic documentation. Thirdly, cost-effectiveness analysis of single-dose delivery versus multi-dose regimens was not conducted. These limitations were mitigated through triangulation across multiple data sources and the use of WHO-standardized assessment tools [8].

### 4.6. Strategic Recommendations

Based on the findings of this analysis, the following recommendations are proposed to accelerate HPV vaccination and advance cervical cancer elimination in West Africa (Table 7). These recommendations align with ongoing WHO and CDC Africa ongoing continental efforts to accelerate cervical cancer elimination across Africa [4,17]. They are organized across five strategic domains: sustaining and expanding coverage, strengthening health systems, ensuring financial sustainability, addressing demand-side barriers, and regional alignment.

The transition to a single-dose schedule adopted by countries based on SAGE recommendations is consistent with the vaccine optimization process, which expand vaccine access and efficiency. Increase access is cost effective and can accelerate progress towards Global Immunization Agenda goal of leaving non one behind while reducing Cervical cancer burden in West African countries [25,26,27,28].

The findings of this study suggest that while some recommendations may be applicable across all the countries, context -specific strategies should be considered when planning and implementing the next phase of HPV vaccination campaigns and cervical cancer elimination in West Africa. Specifically, Ghana could further expand digital monitoring systems, Liberia could strengthen supportive supervision and coordination mechanisms, Côte d’Ivoire could scale outreach approaches for underserved populations, and Sierra Leone could evaluate the cost-effectiveness of its two-round delivery strategy and maintain it in districts with persistent access challenges.

From a long-term perspective, countries should prioritize sustainable domestic financing, link HPV vaccination with cervical cancer screening and treatment services and evaluate the feasibility and cost-effectiveness.

## 5. Conclusions

This study contributes to the growing body of evidence on HPV vaccine implementation in Africa. While previous studies have predominantly focused on single-country experiences, vaccine acceptability, or vaccination coverage, this analysis provides a standardized comparison of HPV vaccine introduction across four West African countries. This study also extends beyond coverage reporting by quantitatively assessing several operational readiness domains, including planning, workforce training, cold-chain preparedness, monitoring systems, and vaccine safety surveillance. Furthermore, it provides early real-world evidence from large-scale MAC campaigns implemented under the WHO-recommended single-dose HPV vaccination strategy. These findings generate practical implementation lessons for countries planning HPV vaccine introduction and scale-up in resource-constrained settings.

The HPV MAC campaigns implemented across Côte d’Ivoire, Ghana, Liberia, and Sierra Leone in 2025 represent an important step in the journey toward cervical cancer elimination in West Africa. Collectively reaching approximately 6.3 million girls with HPV vaccination, these campaigns findings suggest that large-scale adolescent immunization is feasible in resource-limited settings when supported by strong political commitment, effective multisectoral coordination, and adequate technical and financial resources.

The campaigns provide valuable implementation evidence on operational readiness, implementation strategies, equity considerations, and safety monitoring that may inform future HPV vaccination efforts across the African region. The single-dose schedule provides an opportunity to simplify HPV vaccine delivery and expand coverage.

However, sustaining and building upon these achievements will require addressing persistent challenges of vaccine hesitancy, supply constraints, data quality gaps, and financial dependency. With 11 countries in West and Central Africa still without HPV vaccination programmes, there is an opportunity to accelerate HPV vaccination scale-up. Continued investment by governments and partners in HPV vaccination in addition to screening and treatment services, could accelerate progress towards the elimination of cervical cancer as a public health problem in West Africa.

## Figures and Tables

**Table 1:** Analytical Framework and Key Data Sources for HPV MAC Campaign Assessment.

Domain	Key Indicators	Data Sources
Strategic Planning	Campaign plan, microplans, budget, timeline	Country campaign plans, WHO mission reports
Cold Chain & Logistics	Cold chain capacity, equipment functionality, transport	Cold chain assessments, logistics reports
Vaccine Management	Stock availability, wastage rate, distribution	Vaccine stock cards, LMIS data
Capacity Building	Training coverage, cascade completion, competency	Training records, pre/post tests
Advocacy & Communication	SBC materials, media reach, community engagement	IEC materials, media monitoring reports
Supervision & Monitoring	Immunization coverage, Supervision checklists, data quality, feedback	Supervision reports, DHIS2 data
AEFI Surveillance	Reporting rate, investigation, causality	AEFI reports, PowerBI/AVADAR/DHIS2
Coordination	Partner mapping, TWG meetings, resource mobilization	Meeting minutes, partner reports

**Table 2:** Comparative summary of HPV MAC campaign’s administrative coverage and implementation parameters across four West African countries, 2025.

Parameter	Côte d’Ivoire	Ghana	Liberia	Sierra Leone
Campaign period	Apr.–May 2025	Oct.–Dec. 2025	Nov.–Dec. 2025	Nov.–Dec. 2025
Target population	3,535,286	2,226,993	746,523	868,294
Age range targeted	9–18 years	9–14 years	9–14 years	9–14 years
Dose schedule	Single dose	Single dose	Single dose	Single dose
Districts covered	113/113 (100%)	261/261 (100%)	30/30 (100%)	16/16 (100%)
Vaccination sites	17,448	14,000	2334	2900
Vaccinators deployed	12,190	12,000	2500	3200
Girls vaccinated	~3,110,000	~1,880,000	~448,000	868,294
Administrative coverage	88.0%	84.5%	60.0%	100.1%
Districts ≥ 90%coverage	78/113 (69%)	186/261 (71%)	8/30 (27%)	16/16 (100%)
AEFI cases (mild)	155	94	22	43
AEFI rate per 100,000	5.0	5.0	4.9	5.0
Vaccine wastage rate	3.2%	4.1%	2.8%	3.5%
Cold chain functionality	100%	98%	100%	100%

**Table 3:** WHO Operational Readiness Assessment Scores by Country and Domain (%). Minimum Threshold: 80% per domain.

Domain	Côte d’Ivoire	Ghana	Liberia	Sierra Leone	Weight
1. Strategic Planning	100%	93.3%	93.3%	93.3%	12.5%
2. Microplanning & Logistics	100%	100%	100%	100%	12.5%
3. Capacity Building	100%	86.0%	100%	100%	12.5%
4. Cold Chain & Transport	100%	92.3%	100%	100%	12.5%
5. Vaccine Management	100%	66.7%	100%	100%	12.5%
6. Advocacy & Communication	100%	100%	100%	100%	12.5%
7. Supervision & Monitoring	100%	96.2%	100%	100%	12.5%
8. AEFI Surveillance	100%	81.8%	100%	100%	12.5%
Aggregate Score	100.0%	88.9%	99.2%	99.2%	100%

**Table 4:** Training Cascade Summary across Four West African Countries, 2025.

Training Level	Côte d’Ivoire	Ghana	Liberia	Sierra Leone	Total
Master trainers	240	300	50	80	670
District supervisors	1130	2000	250	1920	5300
Frontline vaccinators	12,190	12,000	2500	3200	29,890
Total participants	13,560	14,300	2800	5200	35,860
Training duration	3 days	3 days	5 days	4 days	-
Pre-test score (avg.)	62%	58%	55%	60%	58.8%
Post-test score (avg.)	91%	89%	88%	90%	89.5%

**Table 5:** Communication Channel Deployment across Four West African Countries, 2025.

Communication Channel	Application	Countries Deployed
Radio broadcasting	Nationwide coverage, community radio, talk shows	All four countries
Television programming	National programmes, news segments, PSAs	Ghana, Côte d’Ivoire
Social media platforms	Targeted messaging, influencer engagement	Ghana (advanced), all countries
Community meetings	Direct engagement, dialogue, leader involvement	All four countries
School sensitization	Student sessions, parent–teacher meetings	All four countries
Religious leader engagement	Sermons, announcements, faith-based messaging	All four countries
Traditional authority	Chief announcements, community mobilization	Côte d’Ivoire, Sierra Leone, Liberia
SMS messaging	Direct parent/caregiver messaging	Ghana, Côte d’Ivoire

**Table 6:** Implementation Challenges, Root Cause Analysis, and Mitigation Strategies.

Challenge Category	Description	Root Causes	Mitigation Strategies
Population denominator inaccuracies	Coverage exceeding 100% in some locations	Outdated census data, population mobility, cross-district vaccination	Improved population data systems, community validation
Urban coverage gaps	Lower coverage than rural areas consistently	Mobility, absenteeism, weaker social structures, info gaps	Extended hours, multiple access points, digital communication
Remote area logistics	Distribution and cold chain challenges	Geographic barriers, limited infrastructure	Enhanced mobile teams, extended duration, cold chain equipment
Data reporting delays	Reporting delays affecting monitoring accuracy	Paper-based systems, limited data management capacity	Digital reporting systems, simplified tools, capacity building
Vaccine hesitancy	Misinformation linking HPV vaccine to infertility	Religious misconceptions, social media rumours	Faith leader engagement, proactive risk communication
Financial sustainability	Dependency on external financing	Gavi transition, limited domestic funding	Ministry of finance engagement, domestic resource mobilization

**Table 7:** Strategic Recommendations for Accelerating HPV Vaccination in West Africa.

Recommendation	Key Actions	Priority	Timeline
Transition to routine delivery	Integrate HPV into routine immunization and school health programmes	High	12 months
Scale equity strategies	Targeted microplanning; reach out-of- school girls; HIV+ 2–3 doses	High	Ongoing
Optimise vaccine portfolio	Single dose per Gavi 6.0; VPOP approach	High	By 2026
Enhance data systems	Expand DHIS2; coverage surveys; GIS mapping	High	12–18 months
Community engagement	Risk communication; counter misinformation; local messaging	High	Ongoing
Multisectoral collaboration	Health education partnerships; joint planning	Medium	6–12 months
Long-term financing	Plan for Gavi transition; domestic financing	High	24–36 months
Regional alignment	Africa CDC Roadmap; IA2030; peer learning platforms	Medium	Ongoing

## Data Availability

Public immunization coverage data referenced in this analysis are openly available through WHO/UNICEF Estimates of National Immunization Coverage (WUENIC) at https://www.who.int/teams/immunization-vaccines-and-biologicals/immunization-analysis-and-insights/global-monitoring/immunization-coverage (accessed on 27 July 2026). Multi-age cohort campaign operational data were obtained from the Ministries of Health and national immunization programmes of Côte d’Ivoire, Ghana, Liberia, and Sierra Leone, with support from WHO and partners. Restrictions apply to these datasets as they constitute country-owned programmatic data. Aggregated data may be made available by the corresponding author upon reasonable request and with permission from the originating institutions.
